# Headspace Solid-Phase Microextraction Followed by Gas Chromatography-Mass Spectrometry as a Powerful Analytical Tool for the Discrimination of Truffle Species According to Their Volatiles

**DOI:** 10.3389/fnut.2022.856250

**Published:** 2022-04-25

**Authors:** Natasa P. Kalogiouri, Natalia Manousi, Adamantini Paraskevopoulou, Ioannis Mourtzinos, George A. Zachariadis, Erwin Rosenberg

**Affiliations:** ^1^Laboratory of Analytical Chemistry, Department of Chemistry, School of Sciences, Aristotle University of Thessaloniki, Thessaloniki, Greece; ^2^Institute of Chemical Technologies and Analytics, Vienna University of Technology, Vienna, Austria; ^3^Laboratory of Food Chemistry and Technology, Department of Chemistry, Aristotle University of Thessaloniki, Thessaloniki, Greece; ^4^Department of Food Science and Technology, School of Agriculture, Aristotle University of Thessaloniki, Thessaloniki, Greece

**Keywords:** truffles, SPME (solid-phase microextraction), GC-MS, *Tuber Aestivum*, *Tuber Borchii*, PLS-DA

## Abstract

This study provides the first assessment of the volatile metabolome map of *Tuber Aestivum* and *Tuber Borchii* originating from Greece using headspace solid-phase micro-extraction (HS-SPME) coupled to gas chromatography-mass spectrometry (GC-MS). For the extraction of the volatile fraction, the SPME protocol was optimized after examining the effects of sample mass, extraction temperature, and extraction time using the one-variable at-a-time approach (OVAT). The optimum parameters involved the extraction of 100 mg of homogenized truffle for 45 min at 50°C. Overall, 19 truffle samples were analyzed, and the acquired data were normalized and further processed with chemometrics. Agglomerative hierarchical clustering (HCA) was used to identify the groups of the two species. Partial least squares–discriminant analysis (PLS-DA) was employed to develop a chemometric model that could discriminate the truffles according to the species and reveal characteristic volatile markers for *Tuber* Aestivum and *Tuber* Borchii grown in Greece.

## Introduction

Truffles are below-ground-level growing fungi, and the species belonging to the genus *Tuber* are highly appreciated because of their nutritional value, health benefits, and unique organoleptic properties. Their rich content in minerals, fatty acids, proteins, amino acids etc., and their characteristic aroma and flavor are the key factors responsible for their appreciation as an exclusive food ingredient (1–3).

The quality of the truffles is related to the soil quality, vegetation characteristics and climate of each region. More than 200 species have been discovered in the *Tuber* genus (1). Truffles are mainly grown in Central and South European forests. The most common valuable and extensively appreciated truffle species are *Tuber Melanosporum* (precious black winter truffle), *Tuber Aestivum* (black summer truffle), *Tuber Borchii* (white spring truffle), and *Tuber Magnatum* (precious white autumn truffle). The geographical location where they are grown has significant impact on its volatile attributes (4). Even though they are a rare commodity they are found in relative abundance in Greece. Truffles grown in the Mediterranean region are considered of high quality as the soil and weather conditions, especially in the mountainous areas of north and northwestern Greece, are ideal for the growth of mushrooms of exceptional quality. Recently, particular attention has been given to the volatile analysis of several truffle species originating from France, Spain, Germany, Poland, United Kingdom, Italy, Croatia, Slovenia, Bosnia in Herzegovina, Romania, Bulgaria, Egypt and China (1, 2, 5–8). There is no documentation of truffles grown in Greece, however, despite their superior quality and high economic value. No studies on the volatile fingerprint of *Tuber* species originating from Greece are yet to be established.

The development of analytical methodologies that allow the exploration of the complex volatile composition of truffles is a challenging task. The volatile fingerprint of *Tuber* species has been the object of numerous studies which have employed a variety of techniques (9–14). Gas chromatography (GC) coupled to mass spectrometry (MS) enables the simultaneous identification of several classes of volatiles. Several studies have analyzed the volatile compounds of different truffle species using headspace chromatographic analysis (5, 9, 10), and purge and trap GC-MS (10). Solid-phase microextraction (SPME) is an effective, green, solvent-free, and non-invasive extraction technique used for the analysis of volatiles, integrating sample extraction and analyte enrichment, and can be used for the rapid and direct extraction of analytes from gaseous, liquid, and solid matrices (11–13). SPME is a non-exhaustive technique based on the partition equilibrium of analytes between the extraction phase and the sample matrix. Conventional SPME involves the use of fibers coated with an appropriate stationary phase. The main steps of this technique include: (a) partitioning of analytes between the matrix and the extraction phase, and (b) subsequent desorption of the concentrated analytes into the analytical instrument in a single step (14–16). Head-space solid phase microextraction combined to GC-MS has been used to assess the volatile fingerprint of several truffle species (2, 7, 13, 17, 18). The majority of the studies report the volatiles identified for different analyzed species, and the use of chemometric tools to enhance the conclusions derived from the experimental data and reveal characteristic markers is limited (2, 4). Notably, to the best of the authors' knowledge, there are no reports of the characteristic markers of the *Tuber* species originated from Greece.

Exploratory data analysis is used to improve the understanding and the interpretability of the results. Multivariate techniques are applied to use mathematical models that are able to evaluate all the variables and identify the membership of each sample to its proper class (19). Data exploration reveals hidden information in the chromatographic data in such a form that the analyst obtains a direct representation of it (20). Unsupervised chemometric tools are frequently used to visualize the clustering of the samples based on their similarity, and supervised chemometrics are used for predictive and descriptive modeling to derive patterns among the samples as well as for variable selection (21, 22).

*Tuber Aestivum* and *Tuber Borchii* are highly appreciated in the Mediterranean region. Considering that these species originating from Greece have not been yet analyzed, the objective of this work was to optimize a HS-SPME protocol and assess their volatile metabolome using GC-MS analysis, and further analyse their volatile fingerprint and determine metabolite heterogeneity among the two species using chemometric tools, and establish markers responsible for the discrimination of *Tuber Aestivum* and *Tuber Borchii*. The HS-SPME protocol was optimized, and the chromatographic data were further processed with chemometrics. A Partial Least-Squares Discriminant Analysis (PLS-DA) model was developed and validated to discriminate the samples and to identify characteristic markers for each species.

## Materials and Methods

### Samples

Fresh ascocarps of two truffle species *Tuber Aestivum* (eight samples) and *Tuber Borchii* (eleven samples) were harvested from natural truffle zones in Greece (Prefectures of Ioannina, Achaia, Evoia, and Chalkidiki) during April and July 2021, when truffles mature in this region, and were kindly provided by local suppliers between May and June 2021. As it is known that the expression of vioaltile species is also depending on the maturity of the truffles (23), they were investigated in comparable states of maturity. The truffles were rinsed with tap water and left to dry in an air hood according to Culleré et al. (6). They were stored at −20°C until analysis.

### Instrumentation

The determination of the volatile compounds in truffle samples was conducted using an Agilent 6890 N Gas Chromatograph coupled to an Agilent 5973 K Quadrupole Mass Spectrometric Detector (Palo Alto, CA). The separation of the compounds was performed on a DB-WAX capillary column (60 m × 0.32 mm, 0.25 μm film thickness, Agilent Technologies, Santa Clara, CA) using helium (99.999%) as mobile phase delivered at a flow rate of 1.2 ml min^−1^. The initial oven temperature was 40°C and it was held constant for 5 min. Then the temperature was raised to 240°C (rate 5°C min^−1^) and it was held constant for 5 min. Using this oven program, the separation of the volatile compounds was completed within 50 min. Splitless injection was performed. The following temperatures were adopted: injector temperature; 270°C, MS source; 250°C and MS Quad; 130°C. The compounds were determined in scan mode recording ions with an m/z ratio of 35–350.

### HS-SPME Analysis

An 85 μm Carboxen/Polydimethylsiloxane (CAR/PDMS) fiber attached to a manual SPME fiber holder (Supelco, Bellefonte, PA) was used for the HS-SPME procedure. Conditioning of the fiber was conducted prior to the analysis based on the instructions of the manufacturer. For the extraction of the volatile compounds, an aliquot of truffle (100 mg) was weighted into a 15 ml-glass vial. Extraction of the volatile compounds took place at 50°C within 45 min. After this time span, the fiber was removed from the vial containing the sample and the analytes were desorbed in the injector of the GC-MS instrument for 5 min which previously had been shown to be sufficient for complete desorption, and for the avoidance of sample carry-over. Each experiment was performed in triplicate.

### Optimization of HS-SPME Parameters

Prior to the analysis of the truffle samples, optimization of different parameters that could potentially affect the performance of the HS-SPME method was undertaken. For this purpose, different sample masses (100–500 mg), extraction time spans (15–45 min) and extraction temperatures (30–50°C) were investigated using bulk samples. During optimization the signals of representative compounds belonging to different compound classes (i.e, 2-butanol, 2-butanone, 4-hydroxy-3-hexanone and 1-octen-3-ol) were monitored (1, 5, 7). The peak areas obtained under the individual conditions were normalized with regard to the peak area obtained for each compound under the optimum/selected conditions, and were expressed as percentage.

### Chemometric Analysis

Clustering analysis was employed to divide the samples into clusters according to their similarity criteria. Agglomerative Hierarchical Clustering (HCA) was used to build a hierarchy of clusters, group the samples in different clusters, and visualize the clusters of the samples (24). Partial least squares discriminant analysis (PLS-DA) is a supervised pattern recognition technique used to find the appropriate class for each sample (20). In PLS-DA a mathematical model is built to establish a correlation and classify the samples, knowing the label of each class (25). A PLS-DA prediction model was developed using the MetaboAnalyst 5.0 platform (26) to discover patterns in the chromatographic data of the truffles species, establishing the most important volatile markers responsible for their discrimination. The data table was normalized by dividing the peak area of each individual peak with the sum of all the peak areas of each chromatogram before exporting to HCA and PLS-DA. Pareto scaling was used and the variables were mean-centered (21, 22).

## Results and Discussion

### Optimization Results

#### Evaluation of Sample Mass

**Figure 1:**
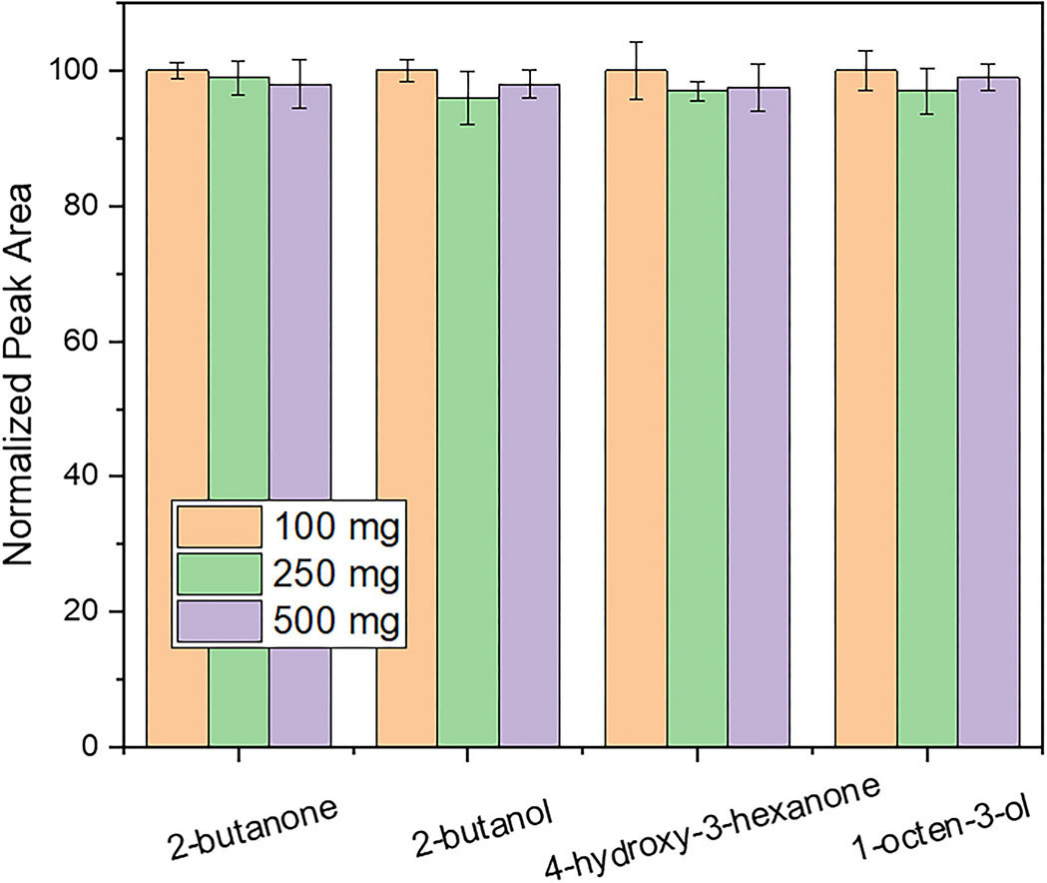
Evaluation of the influence of sample mass on response using an extraction time of 30 min and a desorption time of 5 min. Other parameters as described in the experimental section.

The appropriate amount of truffle sample was initially examined. In principle, the amount of the adsorbed analytes increases by increasing the sample mass. However, unwanted phenomena of fiber overloading might also occur when increasing the amount of sample (27). Thus, the sample mass was investigated between 100 and 500 mg to ensure high method sensitivity in combination with reasonable sample consumption. As shown in Figure 1, an aliquot of 100 mg of truffle sample was sufficient and no increase in the sensitivity of the method was observed upon sample amount increase. Thus, further experiments were conducted with 100 mg of truffle samples.

#### Evaluation of Extraction Temperature

**Figure 2:**
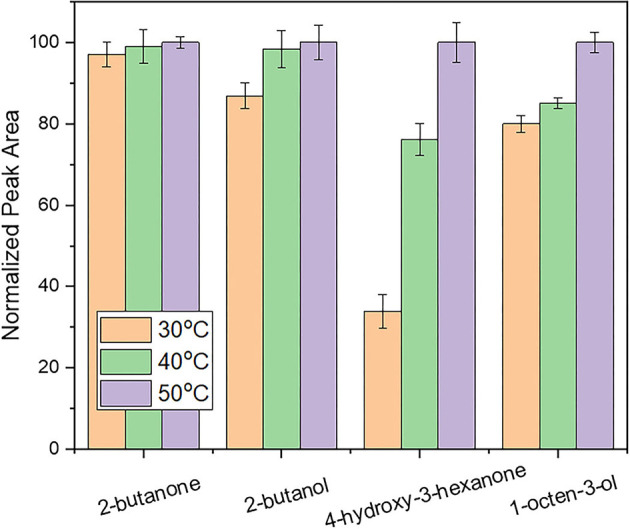
Evaluation of the influence of extraction temperature on response using an extraction time of 30 min and a desorption time of 5 min. Other parameters as described in the experimental section.

Extraction temperature was studied over the range 30 to 50°C. As shown in Figure 2, the intensity of 4-hydroxy-butanone and 1-octen-3-ol was increased by increasing the extraction temperature up to 50°C. This can be attributed to the higher concentrations of analytes that are being released into the headspace by increasing the extraction temperature (30). Further increase of the extraction temperature was not investigated to avoid decomposition of natural products. Thus, an extraction temperature of 50°C was chosen for the HS-SPME procedure.

#### Evaluation of Extraction Time

**Figure 3:**
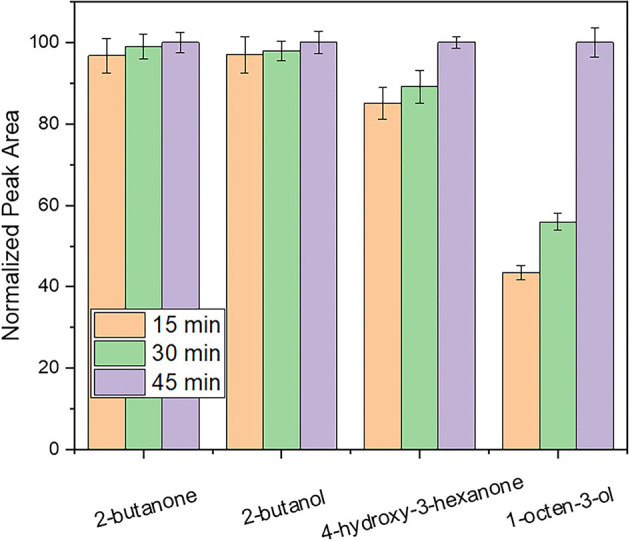
Evaluation of the influence of extraction time on response using a desorption time of 5 min.

After the selection of the optimum sample amount and extraction temperature, the extraction time of the HS-SPME procedure was investigated between 15 and 45 min. This parameter is important to establish the time that is needed for the analytes to reach an equilibrium to ensure the highest sensitivity of the technique (31). As shown in Figure 3, equilibrium was achieved for the less abundant compounds (i.e, 2-butanol, 2-butanone) within 45 min. On the other hand, for the more abundant compounds such as 4-hydroxy-3-hexanone and 1-octen-3-ol the increase of the extraction time resulted in the increase of their intensities even after this time. As is well known from the theory of SPME that equilibration between the fiber coating and the sample headspace is depending (among other factors) on both, sample volatility, partitioning coefficient and also concentration and that equilibration time increases with the latter two factors, the focus of the optimization of this parameter was on the less intense peaks, ensuring that these would be extracted to a high extent. Further increase of the extraction time was not investigated to ensure a rapid extraction process. Thus, 45 min were chosen as the extraction time for further experiments.

### GC-MS Analysis

The optimum extraction parameters were adopted in the subsequent analysis of *Tuber Melanosporum* and *Tuber Aestivum* samples. Overall, 45 compounds were identified using the NIST library (Version 2.0 g, 2011) on the basis of the agreement of the mass spectra as well as their respective retention indices (RI). The peak areas of the identified compounds were normalized after assuming that the sum of the peak areas of all the identified compounds in each sample is equal to 100%. Table 1 presents a list of the identified compounds, their corresponding retention times (RTs), retention index (RI) and their normalized peak areas presented as percentages.

**Table 1:** Volatile compounds identified in *Tuber* Borchii and *Tuber* Aestivum.

				***Tuber* Borchii**	***Tuber* Aestivum**
**RT^a^ (min)**	${\text{RI}}_{\text{experim}.}^{\text{b}}$	${\text{RI}}_{\text{lit}}^{\text{c}}$	**Compound**	**% Peak area**	**% Peak area**
5.762	896	898.0	2-methylfuran	n.d.−0.9%	n.d.– <6%
5.912	904	905.0	2-butanone	n.d.−0.6%	7–30%
6.226	917	921.5	3-methylbutanal	0.7–1.7%	2–36%
6.784	941	932.0	ethanol	0.4–1.8%	1.6–7.3%
7.642	977	989.0	2.3-butanedione	0.3–0.5%	0.9–1.7%
9.251	1035	1031.0	2-butanol	n.d.−0.3%	0.6–26%
10.574	1078	1082.0	hexanal	0.3–0.5%	n.d.−9.7%
10.953	1091	1088.0	2-methyl-2-butenal	n.d.−22%	n.d.−0.1%
11.425	1106	-	unidentified 1	n.d.−0.3%	n.d.−1.3%
11.525	1109	1089.3	2-methyl-1-propanol	n.d.−22%	n.d.−0.12%
12.133	1127	1120.0	3-methylthiophene	0.2–0.5%	n.d.−0.7%
13.892	1182	1185.1	heptanal	0.2–0.4%	n.d.−1.7%
14.442	1199	-	unidentified 2	0.2–0.8%	0.1–2%
14.943	1215	1205.8	2-methyl-1-butanol	1.3–4 %	n.d.−32%
16.073	1253	1263.0	3-methyl-3-buten-1-ol	n.d.−1.4%	n.d.−1.6%
16.158	1256	1254.8	3-octanone	n.d.−29.7%	n.d.−11.2%
18.267	1327	1316.0	3-methyl-2-buten-1-ol	n.d.−1%	n.d.−2%
18.582	1339	1341.0	6-methyl-5-hepten-2-one	0.1–0.3%	n.d.−0.3%
19.168	1359	1351.4	1-hexanol	0.1–0.3%	0.4–6.6%
20.112	1393	1391.5	nonanal	n.d.−0.5%	0.1–0.7%
20.227	1397	1391.9	3-octanol	n.d.−0.6%	0.1–2.7%
20.398	1403	-	unidentified 3	n.d.−0.2%	n.d.−0.05%
20.563	1409	1405.0	2-butoxyethanol	n.d.−0.1%	0.1–0.4%
20.727	1416	-	4-hydroxy-3-hexanone	n.d.−0.4%	n.d.−5%
20.806	1419	1428.0	5-ethylcyclopentene-1-carbaldehyde	n.d.−0.4%	n.d.−5%
21.163	1432	1429.5	2-octenal	0.1–0.2%	0.1–2%
21.321	1438	-	unidentified 4	0.1–0.2%	n.d.−0.4%
21.735	1454	1444.2	1-octen-3-ol	0.4–1.3%	4.6–22%
22.786	1494	1487.9	2-ethyl-1-hexanol	0.8–1.2%	n.d.−0.3%
22.944	1500	1495.9	decanal	0.1–0.2%	n.d.−0.3%
23.115	1507	1498.8	2-acetylfuran	0.1–0.6%	n.d.−1%
23.566	1525	1518.7	benzaldehyde	0.8–1.3%	0.3–0.8%
23.945	1540	1535.9	2-nonenal	n.d.−0.1%	n.d.−0.7%
24.152	1549	1519.6	2-nonanol	n.d.−0.1%	0.1–2%
24.531	1564	1551.6	1-octanol	0.6–1%	0.1–1%
25.975	1623	1610.3	2-octen-1-ol	n.d.−0.1%	0.3–1.5%
26.304	1637	-	unidentified 5	n.d.−0.1%	0.1–0.7%
26.483	1645	1640.7	phenylacetaldehyde	0.2–0.5%	0.1–1.5%
26.912	1663	1656.3	furfuryl alcohol	0.8–2%	0.2–1%
27.27	1679	1664.5	2-methyl-butanoic acid	33–53%	n.d.
28.364	1726	1715.0	3-methylthiopropanol	0.5–1%	0.1–9%
29.3	1768	1754.7	1-decanol	0.3–0.5%	n.d.−0.05%
32.539	1921	1903.7	2-phenylethanol	1–2%	n.d.−0.05%
33.619	1974	1959.3	1-dodecanol	n.d.−0.09%	0.01–0.09%
34.913	2039	2026.1	gamma-nonalactone	0.2–0.3%	n.d.−0.1%

a *RT, retention time*,

b *RI_experim_, experimentally determined retention index*,

c *RI_lit_, retention index obtained from literature sources (28), (29) and from PubChem. n.d., not detected*.

The characteristic volatiles that are responsible for the unique aroma of the truffles belonged mainly to the classes of aldehydes, alcohols, and ketones (32). According to Table 1, 3-methyl-butanal (2–36%) was the most abundant compound and is responsible, according to the descriptions in the literature, for the “sulfurous” and “animal” odor, and has been reported as a characteristic volatile of black truffle species and characteristic marker of truffle degradation and product spoilage produced by axenic cultures of truffle mycelium, fungal and bacterial phyla (5, 8). The second most abundant compound to be identified in *Tuber Aestivum* was 2-methyl-1-butanol at a percentage of up to 32%. It is a naturally occurring alcohol responsible for the ethereal type odor that has already been determined in black truffles (5). Furthermore, 2-butanone was identified and determined within the range between 7 and 30% which has been previously reported to vary among *Tuber Aestivum* ascorcaps (8). Octen-3-ol, imparting “mushroom, earthy herbal, woody, green” odor notes (4, 33), was determined over the range 5–22%. Its presence in black truffle species has been associated with the earthy and dusty aroma of the product. In addition, 3-octanone (<11.2%) and ethanol (2–7%) that are responsible for “mushroom/herbal” and “alcoholic” notes (34), respectively, and their high abundance in black truffle species has already been reported (5, 8, 35) were also detected.

In *Tuber Borchii*, the most abundant compound identified was 2-methyl-butanoic acid within the range 33–53%, and this has already been reported to contribute to truffle aroma (18, 36). 2-methyl-butanoic acid is responsible for the pervasive, cheesy, sweaty odor. The percentages of 2-methyl-1-propanol and 2-methyl-2-butenal were also high up to 22%, for both compounds. 2-methyl-1-propanol is associated with descriptors like “sweet” and “musty”, and 2-methyl-butenal is responsible for the “green” and “fruity” odor. The presence of 2-methyl-1-propanol and 2-methyl-2-butenal in *Tuber* Borchii has been previously reported by D'Auria et al. (37), as well.

As regards the rest of the identified compounds, 2-methyl-1-butanol and 2-butanol, have been identified in black truffle species (5). The presence of dodecanol, phenylethanol, 2-methyl-butanoic acid, 2-octen-1-ol, 1-octanol, benzaldehyde, 1-hexanol, heptanal, hexanal, and 2,3-butanedione, in black truffles has been reported by Choo et al. (4). Nonanal, decanal, 2-nonenal, 2-methyl furan, 2-octenal, 2-ethyl-1-hexanol, 6-methyl-5-hepten-2-one, 3-octanone, and 3-octanol have been identified in the white truffle species *Tuber Magnatum Pico* originating from Italy, as well (18, 36).

### Chemometrics

#### Cluster Analysis

**Figure 4:**
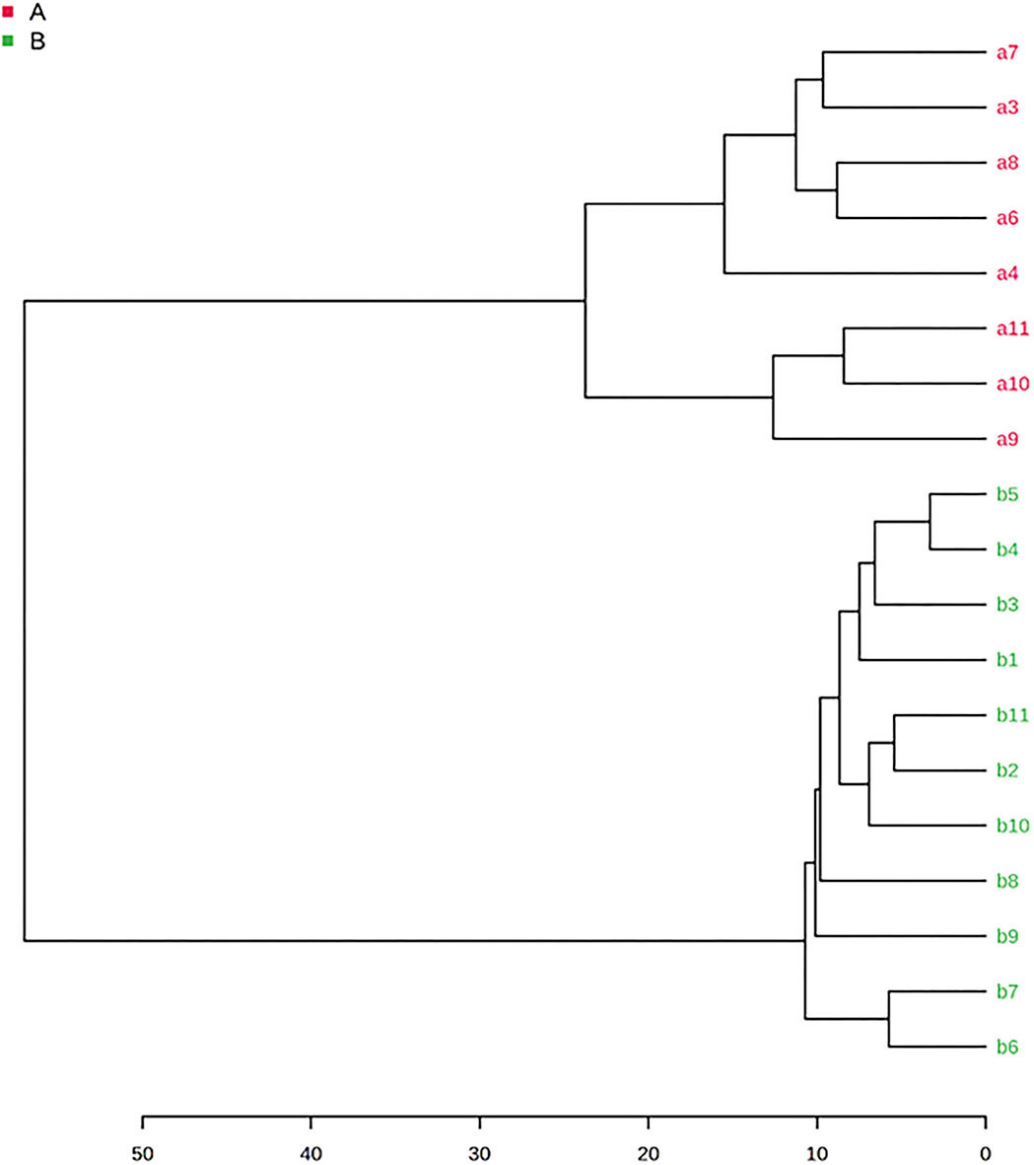
HCA dendrogram of *Tuber* Aestivum and *Tuber* Borchii (Samples belonging to the *Tuber* Aestivum species are shown in red color, and the samples belonging to *Tuber* Borchii species are shown in green color).

HCA was used to develop a tree diagram and visualize the classes of the samples. The dendrogram in Figure 4 shows the clustering of the samples into two separate groups. The truffles were distributed forming two major clusters, one cluster for the samples belonging to the *Tuber Aestivum* species shown in red color, and one cluster for the samples belonging to *Tuber Borchii* species, shown in green color.

#### PLS-DA

**Figure 5:**
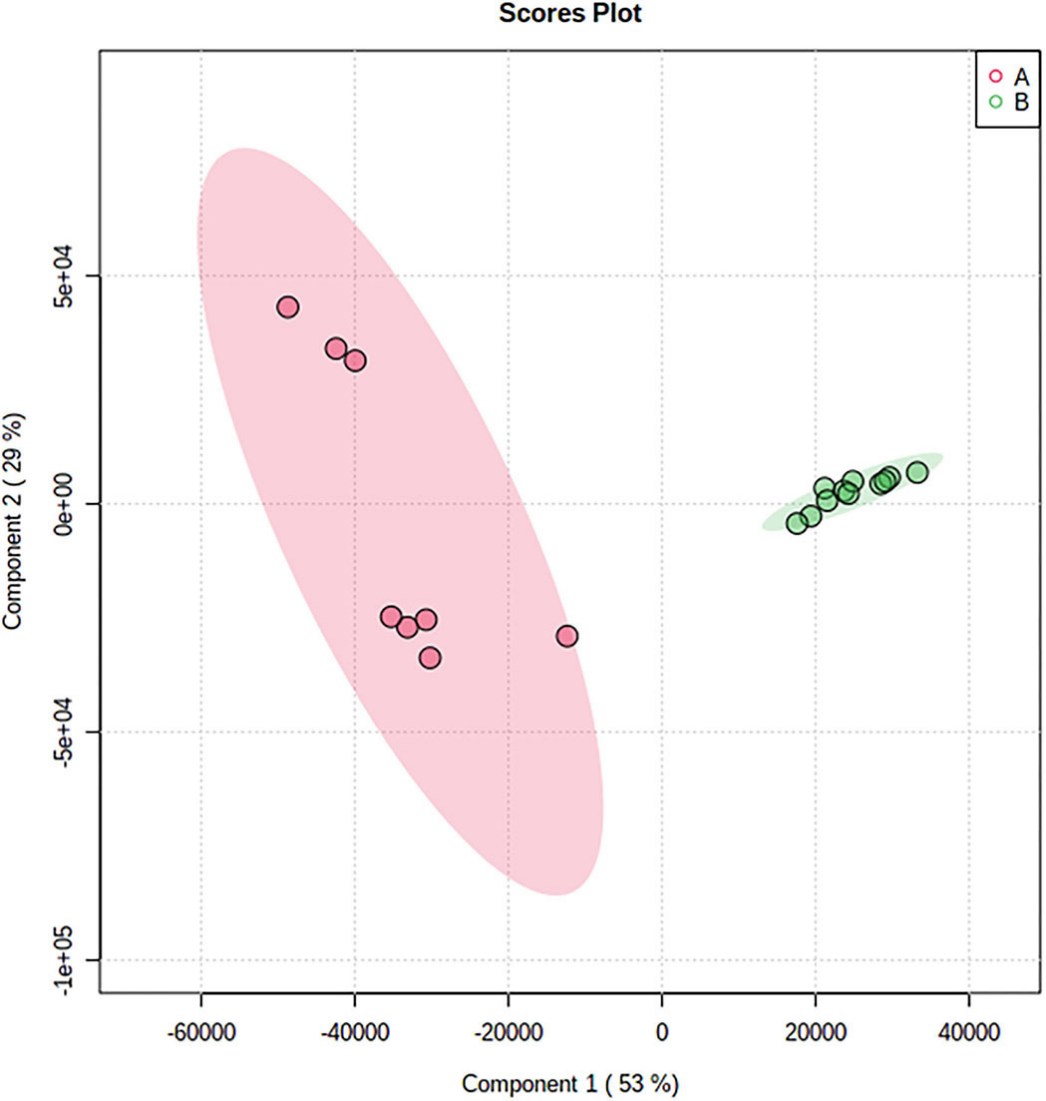
PLS-DA score plot showing the discrimination of the samples according to their species; *Tuber* Aestivum samples are grouped together in the red ellipse, and *Tuber* Borchii samples are grouped together in the green ellipse.

**Figure 6:**
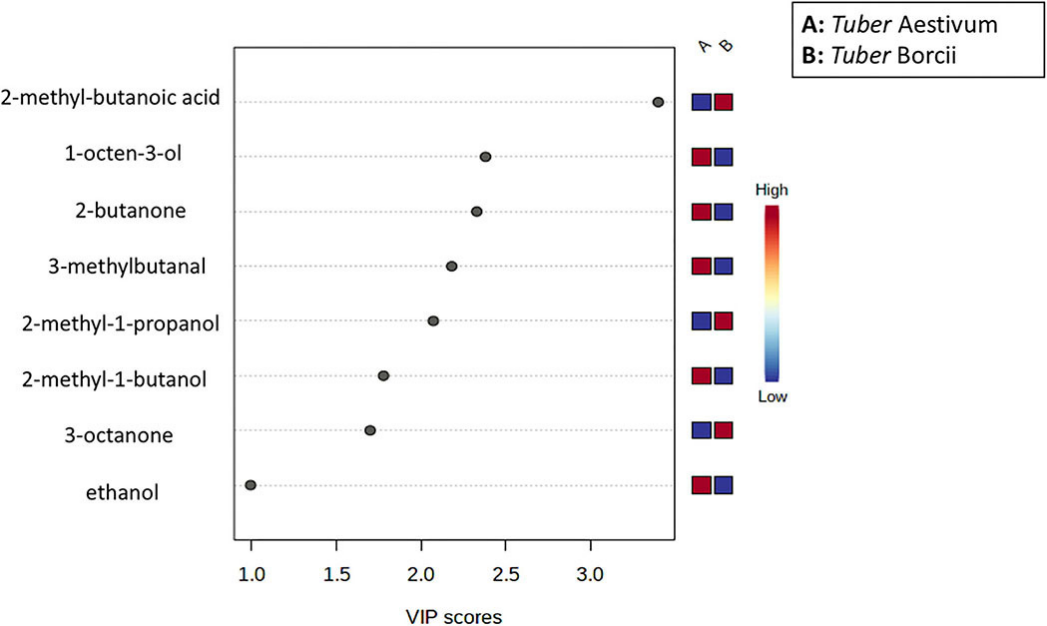
VIP score showing the most important features causing greater variation in the PLS-DA model; The features causing higher variation are shown in red color, and those showing the lowest variation are shown in blue for each group (A: *Tuber* Aestivun, B: *Tuber* Borchii).

To assess the volatile variations within *Tuber Aestivum* and *Tuber Borchii* and specify markers for each species a PLS-DA model was developed in the MetaboAnalyst platform. The PLS-DA model successively grouped the samples according to the species with an explained variance of 82% in the first two dimensions. The PLS-DA scores plot shows a clear discrimination between the two species, as it is shown in Figure 5. Specifically, *Tuber Borchii* specimens were positioned separately in the green ellipse, and *Tuber Aestivum specimens* were grouped in the red ellipse, and the colored areas around the samples represent the 95% confidence region of replicates. In an attempt to evaluate the significance of each variable in projection (VIP) was used to build the PLS-DA model, VIP scores were calculated to identify the most significant features responsible for the grouping of the truffle species. The VIP scores estimate the significance of each variable in projection, showing their contribution in the final model, using the cut-off value of above 0.83 according to Mehmood et al. (22). According to the VIP scores, 2-methyl-1-butanoic acid, 2-methyl-1-propanol, and 3-octanone cause greater variation in the *Tuber Borchii* species, while 1-octen-3-ol, 2-butanone, 3-methyl-butanal, 2-methyl-1-butanol, and ethanol are characteristic volatiles of the *Tuber Aestivum* species, as it is shown in Figure 6.

**Figure 7:**
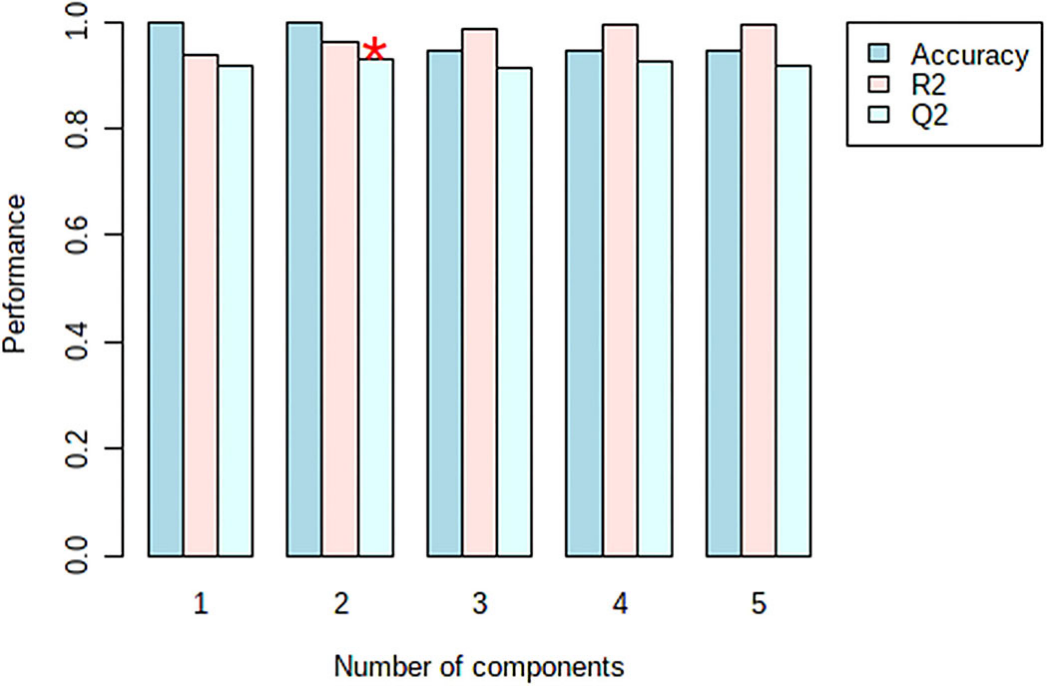
Cross validation parameters of the developed PLS-PDA model with the prediction error measure: accuracy, R2, Q2 (The accuracy = 1.0, was obtained from the second component shown with asterisk).

The model was validated using the Leave-One-Out Cross-Validation method (LOOCV) using five components (Figure 7). According to the validation results, the goodness of fit (*R*^2^ = 0.96), and the predictability of the model (*Q*^2^ = 0.94) confirm the good performance of the prediction model. For the permutation test statistics, 100 random permutations were calculated and the results showed that the truffle samples differ statistically (with one sample *t*-test with *p* < 0.01) (38).

## Conclusions

Nineteen samples of truffles belonging to the species *Tuber Aestivum* and *Tuber Borchii* grown in Greece were analyzed by HS-SPME coupled with GC-MS to assess their volatile fingerprint. The SPME protocol was optimized after evaluating the effects of sample mass, extraction temperature and extraction time with the OVAT approach. The optimum parameters involved the extraction of 100 mg of truffle, at 50°C for 45 min. In total, 45 volatile compounds were detected and further processed with chemometrics. 2-Methyl-butanoic acid, 2-methyl-1-propanol, and 2-methyl-2-butenal were identified as the most abundant volatiles in *Tuber Borchii*, while in *Tuber Aestivum*, the most abundant volatile compounds were 3-methyl-butanal, 2-methyl-1-butanol, 2-butanone, 1-octen-3-ol, and ethanol. An HCA dendrogram was developed showing the clustering of two major groups according to the *Tuber* species. A PLS-DA chemometric model was developed and was able to group the truffles according to their species with 82% of explained variance. The findings of this research clearly demonstrate that HS-SPME coupled to chemometrics can effectively be applied in the discrimination of different Tuber species.

## Data Availability Statement

The original contributions presented in the study are included in the article/supplementary materials, further inquiries can be directed to the corresponding author.

## Author Contributions

NK, NM, and IM designed the study. NK and NM conceived the experiment design, performed the experiments, and drafted the manuscript. NK, NM, and AP analyzed and interpreted the data. NK performed the chemometric and statistical analyses. GZ, AP, IM, and ER supervised the study. All authors provided critical revisions and approved the final version of the manuscript.

## Conflict of Interest

The authors declare that the research was conducted in the absence of any commercial or financial relationships that could be construed as a potential conflict of interest.

## Publisher's Note

All claims expressed in this article are solely those of the authors and do not necessarily represent those of their affiliated organizations, or those of the publisher, the editors and the reviewers. Any product that may be evaluated in this article, or claim that may be made by its manufacturer, is not guaranteed or endorsed by the publisher.
